# Adverse drug reactions and polypharmacy in cardiac patients

**DOI:** 10.1186/2050-6511-13-S1-A39

**Published:** 2012-09-17

**Authors:** Snežana Mugoša, Zoran Todorović, Majda Šahman-Zaimović

**Affiliations:** 1 Department of Pharmacotherapy, Faculty of Pharmacy, University of Montenegro, 81000 Podgorica, Montenegro; 2 Institute of Pharmacology, Clinical Pharmacology and Toxicology, Faculty of Medicine, University of Belgrade, 11000 Belgrade, Serbia; 3 Medicines and Medical Devices Agency of Montenegro, 81000 Podgorica, Montenegro

## Background

Adverse drug reactions (ADR) appear more frequently then what is actually reported and registered. The aim was to establish an intensive monitoring system and to analyze ADR in hospitalized patients.

## Methods

The prospective study covered 200 patients hospitalized in the Cardiology Center, Clinical Center of Montenegro. ADR were recorded in the following way: patients were interviewed on the basis of a symptoms list and any signs which could point to eventual ADR. Secondly, lab tests and other available parameters were monitored.

## Results

At the time when interviews took place, patients received on average 8.0 ± 2.6 medicines (2–17). In total, 67 patients (33%) had 75 ADR. Twenty one ADR (28%) are classified as serious. Fifty four ADR resulted in the recovery of the patient (72%), eight had as an outcome prolonged hospitalization (11%), another eight were life threatening (11%), while five ADR (6%) were the cause of the hospitalization. The most frequent ADR which had as an outcome admission to hospital were caused by digoxin (40%), prolonged hospital stay by furosemide (38%), while the most frequent registered ADR which were life threatening were caused by streptokinase (50%).

## Conclusions

Monitoring ADR in patients using cardiovascular drugs is a matter of importance since this class of medicines is usually used by elderly patients with critical conditions and accompanying diseases. Considering increased use of cardiovascular drugs and limitations in pre-marketing trials for drug safety evaluation, post marketing evaluation of adverse drug reactions induced by this class of medicinal products seems necessary. Additional educational efforts could affirm the rationalization of the pharmacotherapy.

